# Supplementary material for: Low-energy electrons transform the nimorazole molecule into a radiosensitiser
Source: Nat Commun. 2019 Jun 3;10:2388. doi: 10.1038/s41467-019-10340-8 (PMC6546713; doi:10.1038/s41467-019-10340-8)
Supplement: Supplementary file 1 — Supplementary Information [file 41467_2019_10340_MOESM1_ESM.pdf]

Supplementary Information to

Low-energy electrons transform the nimorazole molecule into a radiosensitiser

Rebecca Meißner[1,2], Jaroslav Kočišek[3], Linda Feketeová[4], Juraj Fedor[3], Michal Fárník[3], Paulo Limão-Vieira[2], Eugen Illenberger[1,5], Stephan Denifl[1]*

[1]Institut für Ionenphysik und Angewandte Physik and Center for Biomolecular Sciences Innsbruck, Leopold-Franzens Universität Innsbruck, Technikerstrasse 25, A-6020 Innsbruck, Austria

[2]Atomic and Molecular Collisions Laboratory, CEFITEC, Department of Physics, Universidade NOVA de Lisboa, 2829-516 Caparica, Portugal

[3]J. Heyrovský Institute of Physical Chemistry v.v.i., The Czech Academy of Sciences, Dolejškova 3, 18223 Prague, Czech Republic

[4]Université de Lyon; Université Claude Bernard Lyon1; Institut de Physique Nucléaire de Lyon, CNRS/IN2P3 UMR 5822, 69622 Villeurbanne Cedex, France

[5]Institut für Chemie und Biochemie-Physikalische und Theoretische Chemie, Freie Universität Berlin, Takustrasse 3, 14195 Berlin, Germany

*Corresponding author. E-Mail: Stephan.Denifl@uibk.ac.at

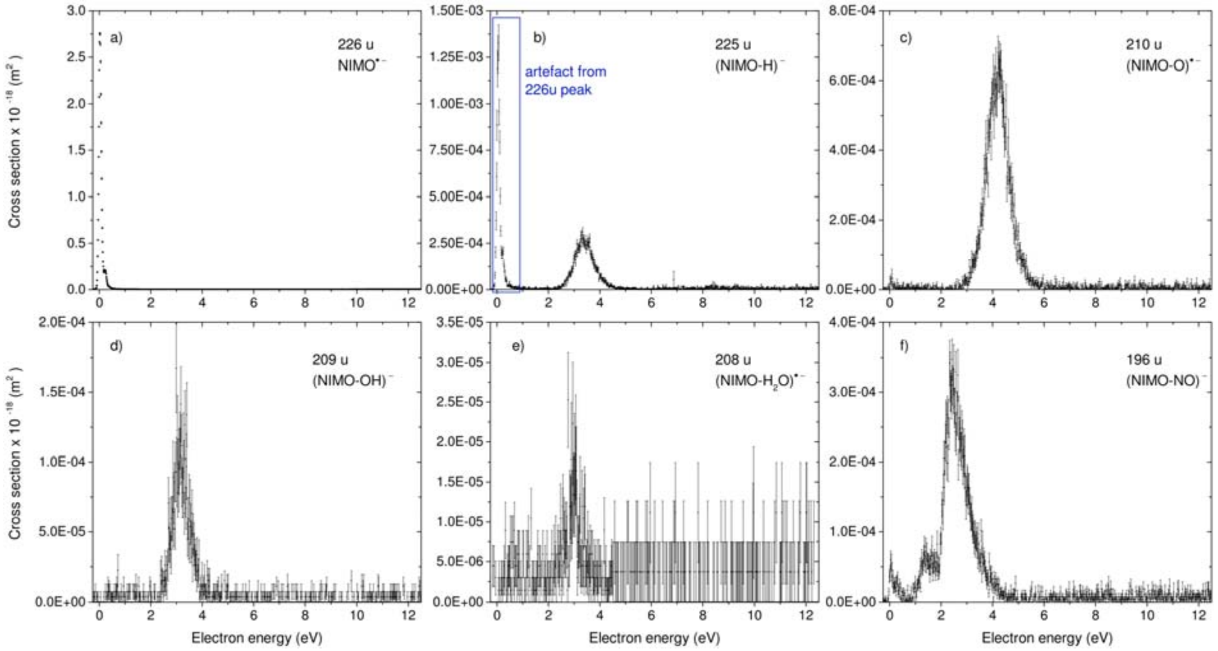

Supplementary Figure 1. Cross-section as function of the initial electron energy. Cross-section for the formation of the nimorazole radical anion (mass 226 u) and fragment anions with masses between 225 u, $(NIMO – H)^{–}$, and 196 u, $(NIMO – NO)^{–}$, in the electron energy range between ~0 eV and 12 eV. The black dots represent the experimentally determined cross-section values as a function of the electron kinetic energy. Statistical error margins are included for each data point and refer to the standard error of the mean, see data analysis section for details in the main paper. The low energy feature in the $(NIMO – H)^{–}$ ion yield can be ascribed to an artefact from the finite mass resolution of the used quadrupole mass spectrometer and the far higher cross-section for formation of $NIMO^{•–}$, which contaminates here $(NIMO – H)^{–}$. Source data are provided as a Source Data file.

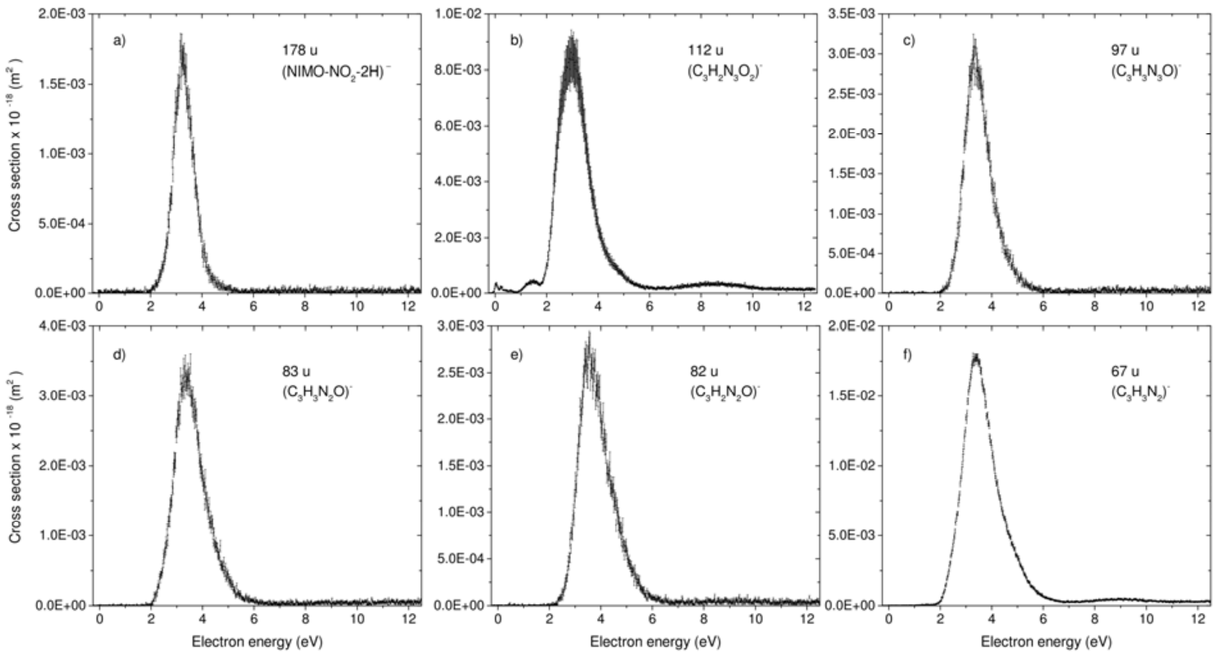

Supplementary Figure 2. Cross-section as function of the initial electron energy. DEA Cross-section for the formation of fragment anions with masses between 178 u, (NIMO – NO_2 – 2H)$^-$, and 67 u, ($C_3H_3N_2$)$^-$, in the electron energy range between ~0 eV and 12 eV. The black dots represent the experimentally determined cross-section values as a function of the electron kinetic energy. Statistical error margins are included for each data point and refer to the standard error of the mean, see data analysis section for details in the main paper. Source data are provided as a Source Data file.

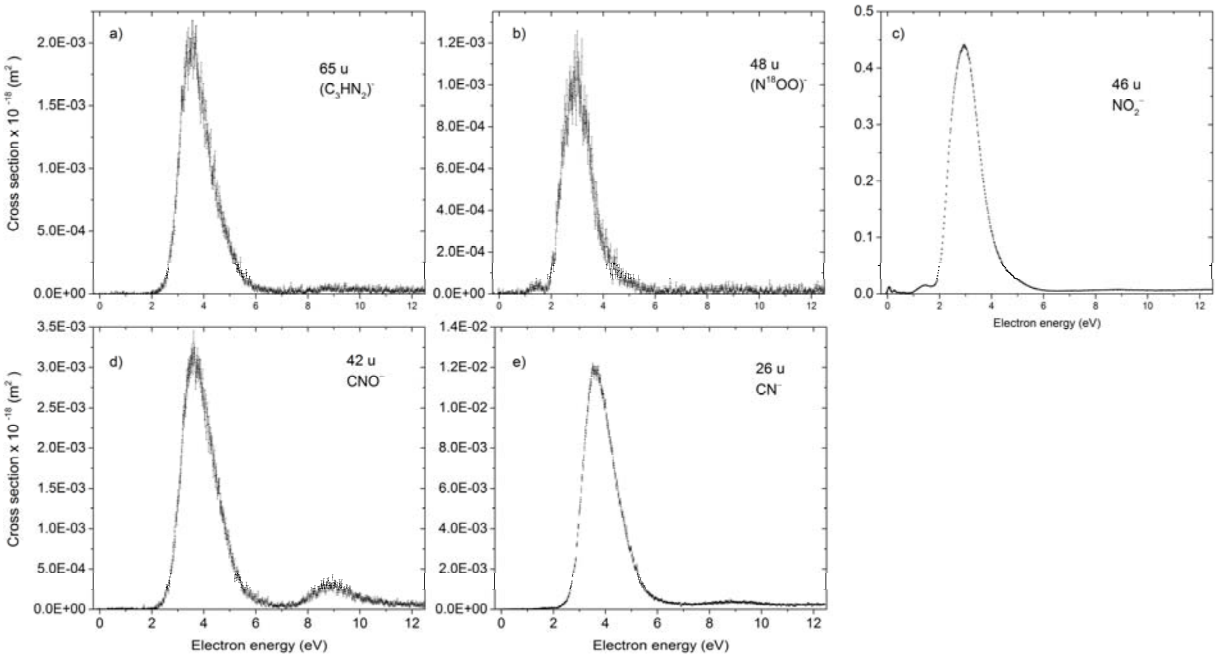

Supplementary Figure 3. Cross-section as function of the initial electron energy. DEA Cross-section for the formation of fragment anions with masses between 65 u, $(C_3HN_2)^-$, and 26 u, $(CN)^-$, in the electron energy range between ~0 eV and 12 eV. The black dots represent the experimentally determined cross-section values as a function of the electron kinetic energy. Statistical error margins are included for each data point and refer to the standard error of the mean, see data analysis section for details in the main paper. Source data are provided as a Source Data file.

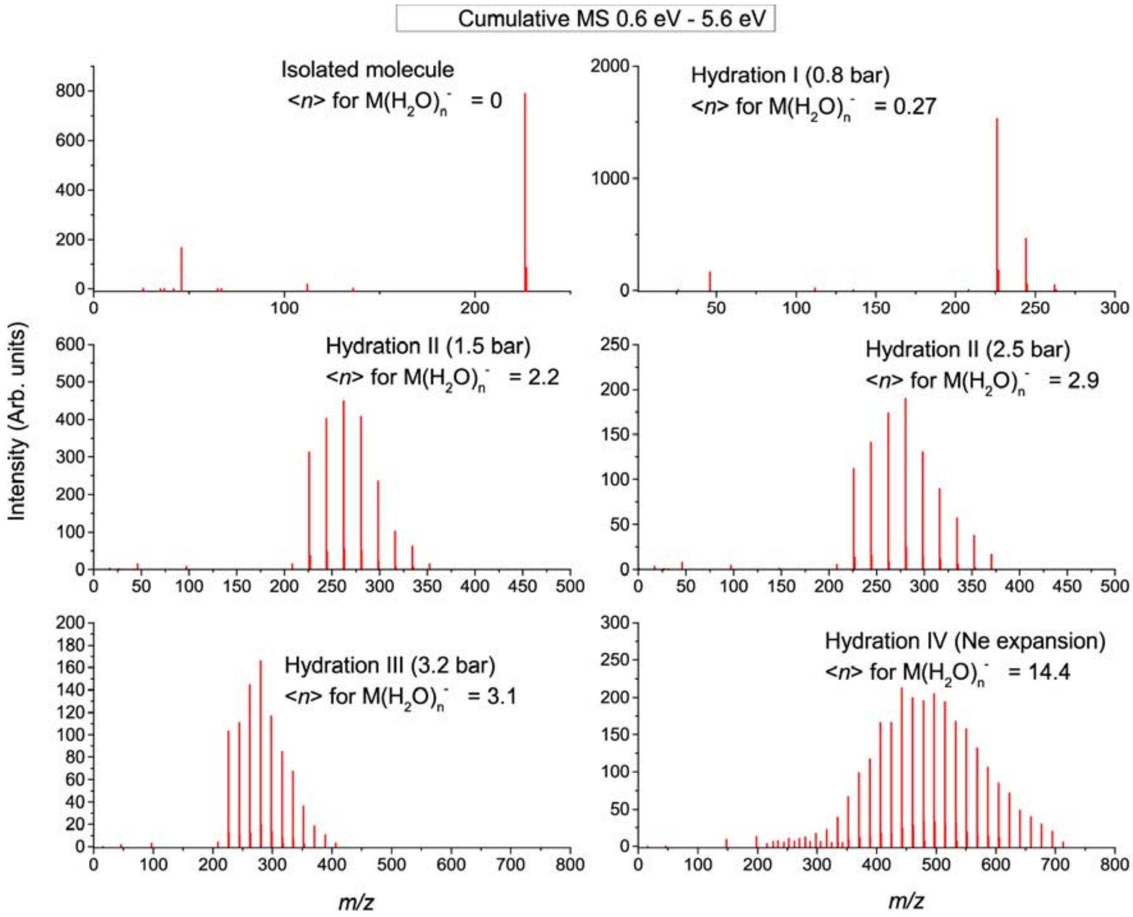

Supplementary Figure 4. Cumulative mass spectra for different hydration conditions. The cumulative mass spectra are used for calculation of intensity ratio of NO_2^- to $NIMO(H_2O)_n^-$ ion signal and mean number of water molecules <n> in the mixed cluster $NIMO(H_2O)_n^-$, which are plotted in the Figure 3. Source data are provided as a Source Data file.

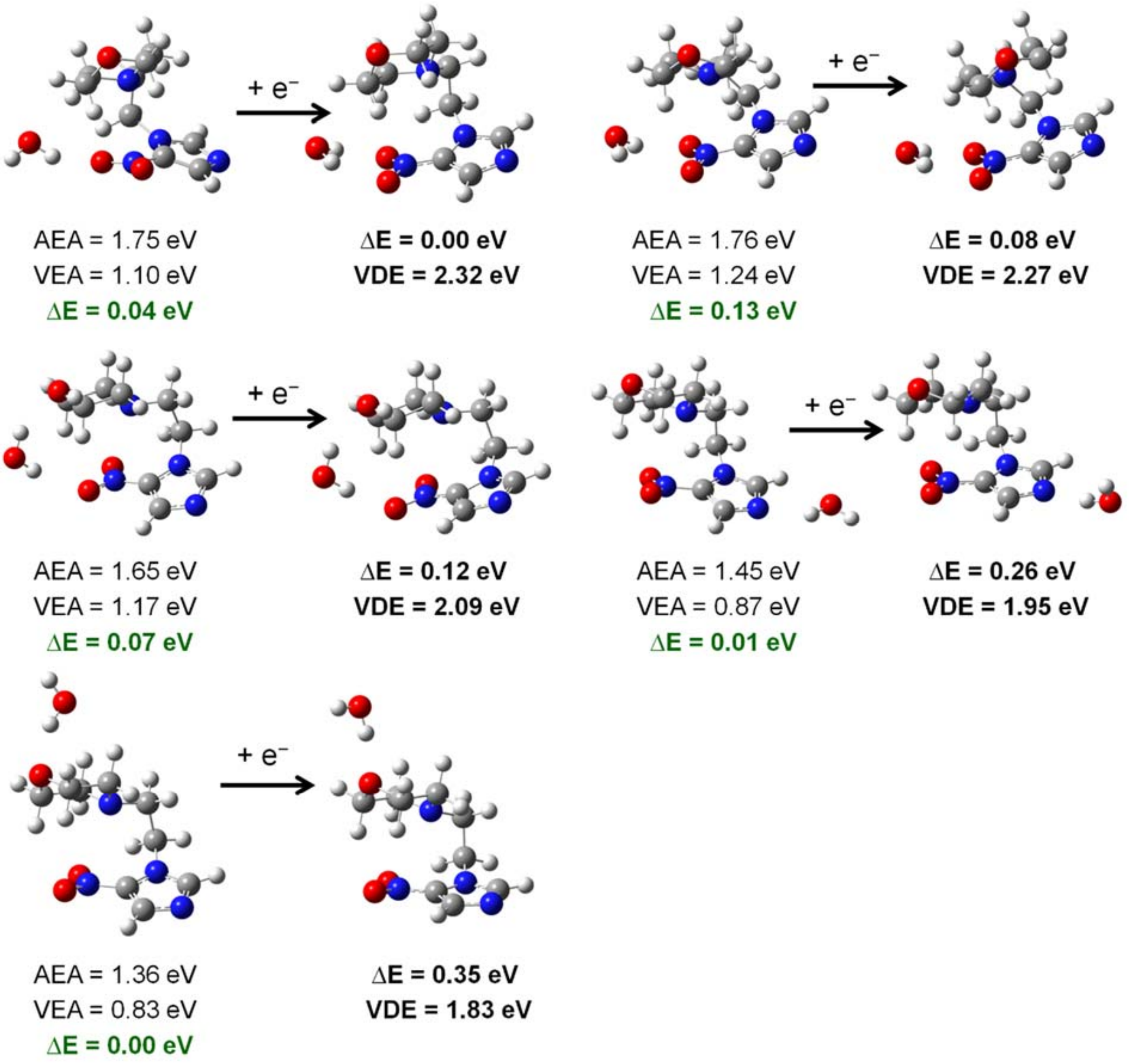

Supplementary Figure 5. Nimorazole hydrated by one water molecule. M062x/6-31+G(d,p) optimized structures of neutral and anionic NIMO hydrated by one water molecule. Adiabatic electron affinities (AEA), vertical electron affinities (VEA) are shown below the neutral structures together with relative energies in green. The relative energies of anionic structures and corresponding vertical detachment energies (VDE) are shown below the structures of the anions. Colour coding: Carbon (grey), hydrogen (white), nitrogen (blue), oxygen (red).

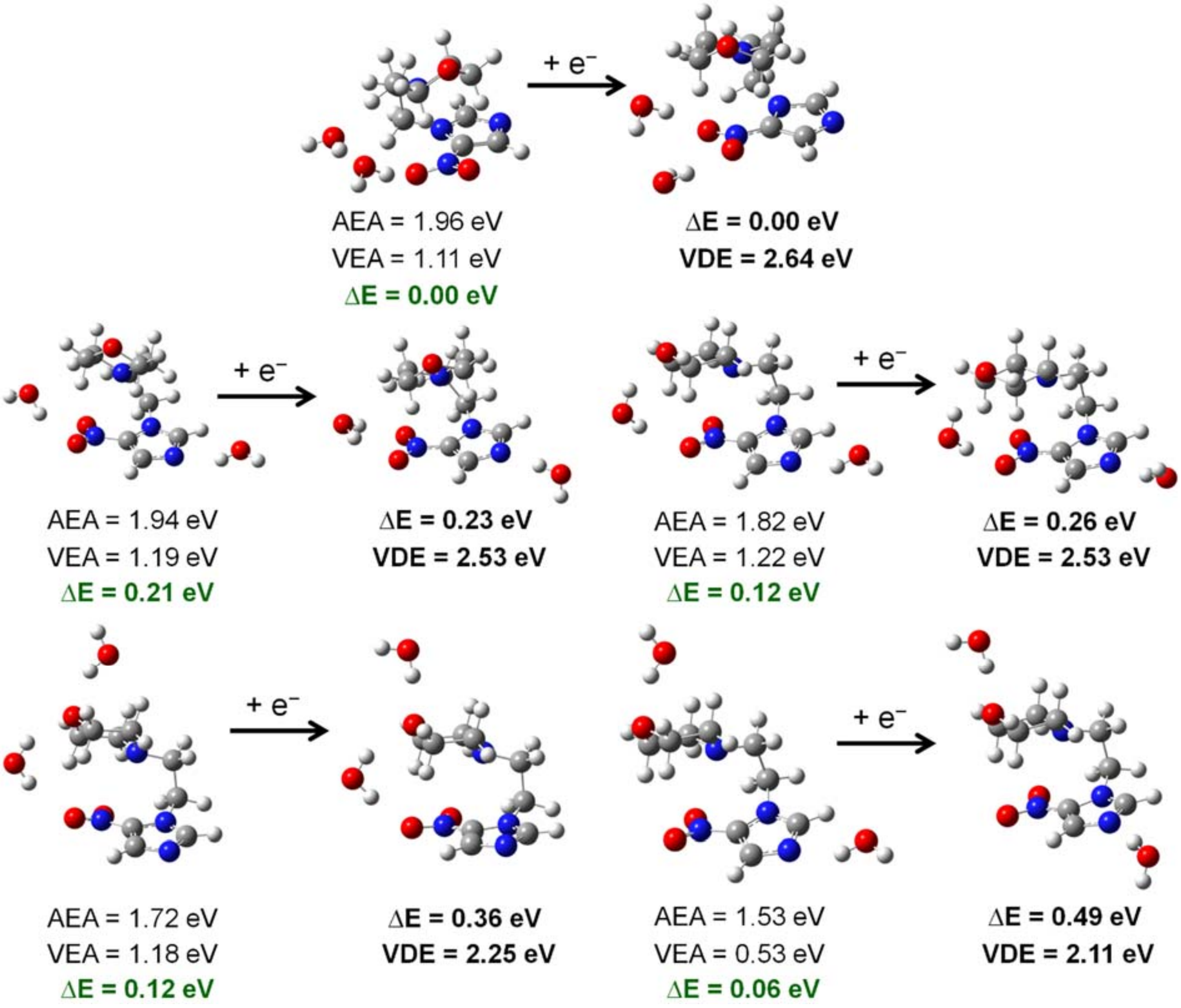

Supplementary Figure 6. Nimorazole hydrated by two water molecules. M062x/6-31+G(d,p) optimized structures of neutral and anionic NIMO hydrated by two water molecules. Adiabatic electron affinities (AEA), vertical electron affinities (VEA) are shown below the neutral structures together with relative energies in green. The relative energies of anionic structures and corresponding vertical detachment energies (VDE) are shown below the structures of the anions. Colour coding: Carbon (grey), hydrogen (white), nitrogen (blue), oxygen (red).
